# Saliva phosphorylated tau concentration is not associated with Alzheimer’s disease, cerebrospinal fluid or blood biomarkers

**DOI:** 10.3389/fnins.2025.1718237

**Published:** 2025-12-02

**Authors:** Helena Sophia Gleerup, Federica Sanna, Srinivas Koutarapu, Juan Lantero-Rodriguez, Laia Montoliu-Gaya, Jörg Hanrieder, Gunnar Brinkmalm, Thomas K. Karikari, Joel Simren, Peter Høgh, Kaj Blennow, Steen Gregers Hasselbalch, Henrik Zetterberg, Nicholas J. Ashton, Anja Hviid Simonsen

**Affiliations:** 1 Department of Neurology, Danish Dementia Research Centre (DDRC), Copenhagen University Hospital, Rigshospitalet, Copenhagen, Denmark; 2 Department of Psychiatry and Neurochemistry, Institute of Neuroscience and Physiology, The Sahlgrenska Academy at the University of Gothenburg, Mölndal, Sweden; 3 Università degli Studi di Cagliari, Cagliari, Italy; 4 Department of Pathology and Immunology, Washington University School of Medicine, St. Louis, MO, United States; 5 Clinical Neurochemistry Laboratory, Sahlgrenska University Hospital, Mölndal, Sweden; 6 Department of Neurodegenerative Disease, UCL Institute of Neurology, London, United Kingdom; 7 Regional Dementia Research Centre, Region Zealand, Roskilde Hospital, University of Copenhagen, Roskilde, Denmark; 8 Department of Clinical Medicine, Faculty of Health and Medical Science, University of Copenhagen, Copenhagen, Denmark; 9 UK Dementia Research Institute at UCL, London, United Kingdom; 10 Hong Kong Centre for Neurodegenerative Diseases, InnoHK, Hong Kong, China; 11 Wisconsin Alzheimer’s Disease Research Centre, University of Wisconsin School of Medicine and Public Health, University of Wisconsin-Madison, Madison, WI, United States; 12 Banner Alzheimer's Institute, University of Arizona, Phoenix, AZ, United States; 13 Banner Sun Health Research Institute, Sun City, AZ, United States

**Keywords:** Alzheimer’s disease, biomarkers, saliva, plasma, CSF, mass spectrometry, immunohistochemistry

## Abstract

**Objective:**

One of the most challenging aims of the scientific community in the last decade, is to find an easily accessible matrix in which neurodegeneration-related biomarkers can be measured and used to diagnose Alzheimer’s disease (AD) *in vivo*. Blood biomarkers have led the way in this regard, specifically, phosphorylated tau (p-tau) which demonstrates excellent diagnostic and prognostic properties. The recent success of the blood biomarkers for AD pathophysiology poses a new question – can p-tau be measured in other peripheral and even more accessible biofluids, and do they have relation to disease? Saliva contains biomarkers linked to neurodegeneration and it has been proposed as a potential sample type that would be minimally invasive to collect for this purpose.

**Methods:**

In this study, we confirmed the presence of several p-tau species in saliva fluid and saliva gland tissue by Immunoprecipitation-Mass spectrometry (IP-MS) and immunohistochemistry, respectively. Furthermore, we measured saliva and plasma p-tau181 concentrations in 125 memory clinic participants, using ultrasensitive Single molecule array (Simoa) technology.

**Results:**

Despite a weak correlation between saliva p-tau181 and CSF t-tau (rho = 0.13, *p* < 0.01), there were no significant differences in saliva p-tau181 concentration between the different clinical groups and the healthy controls.

**Interpretation:**

For this reason, we conclude that saliva p-tau181 is not acceptable as a biomarker for AD.

## 1 Introduction

Alzheimer’s disease (AD) is the most common cause of dementia and affects approximately 55 million people worldwide (Long et al., 2023). Easily accessible and accurate biomarkers could be employed in primary care to increase the confidence of an AD diagnosis or rapidly identify another neurodegenerative disorder causing the underlying symptomatology, which would require more detailed investigation at a memory clinic. In addition, such biomarkers could be used to facilitate therapeutic trials targeting AD pathology, as accurate and easily accessible biomarkers are needed to successfully recruit individuals.

Currently, only histopathological examination of brain tissue allows for the conclusive diagnosis of AD, highlighting the two main hallmarks of the disease: amyloid-*β* (Aβ) plaques and tau neurofibrillary tangles (NFT). Yet, clinical assessment, supported by positron emission tomography (PET) or cerebrospinal fluid (CSF) biomarkers, is a strategy that accurately reveals AD pathophysiology. PET measurements of Aβ and tau pathologies have been validated against the gold-standard of histopathological examination (Ikonomovic et al., 2016; Fleisher et al., 2020). CSF measures of Aβ42/40, total tau (t-tau) and phosphorylated tau (p-tau) also accurately depict AD pathology as measured by PET and histopathological examination (Mattsson-Carlgren et al., 2022; Palmqvist et al., 2015). However, high costs and adverse effects of PET, and the invasiveness of a lumbar puncture, are disadvantages which restrict the application of these techniques to evaluate suspected AD, to monitor the longitudinal change or be widely employed as population screening in disease-modifying therapy recruitment. These limitations have driven efforts to find a non-invasive, inexpensive, and accessible methods to detect specific pathologies.

Blood-based measures for p-tau, t-tau, neurofilament (NfL) and Aβ have been proposed to be this non-invasive biomarker for AD (Gleerup et al., 2025). However, plasma t-tau has limited diagnostic utility due to large overlaps between diagnostics groups (Simrén et al., 2021). NfL, in serum or plasma, a measure of axonal injury, is increased in most neurodegenerative disorders and acute neurological disorders (Ashton et al., 2021; Simonsen et al., 2023). Therefore, its strength lies in being a non-specific measure of neurodegeneration rather than one for AD pathology. Plasma measures of Aβ42/40 have shown more promise in AD but the more sensitive and accurate techniques demonstrate small fold-changes (10%) in comparison to CSF Aβ42/40 (50%) when classifying Aβ PET positive and Aβ PET negative individuals (Schindler et al., 2019; Keshavan et al., 2021). Thus, plasma Aβ42/40 is highly sensitive to variations in biomarker measures (Benedet et al., 2021). More prominently, the detection of p-tau in blood has been revealed to be specifically increased in AD, and is validated against Aβ and tau PET, CSF biomarkers as well as neuropathological diagnosis. Plasma p-tau is now being widely deployed in primary and secondary care as a supportive tool for the diagnose of AD (Palmqvist et al., 2025). Especially, p-tau217 has shown remarkable diagnostic performance when compared to other biomarkers, distinguishing AD from other neurodegenerative diseases. Furthermore, p-tau217 may detect AD pathology at an earlier disease stage and have shown a stronger dynamic range, making it a promising biomarker candidate for both clinical and research applications (Hansson, 2021; Palmqvist et al., 2020; Janelidze et al., 2020).

Recent studies have also highlighted these biomarkers in saliva, suggesting their use for neurodegenerative disorders (Gleerup et al., 2019; Ng et al., 2024). The sampling process for saliva is non-invasive and non-expensive, which could be replicated several times and regularly with no need for the expertise of a venepuncture (Ashton et al., 2019; Gleerup et al., 2021; Gleerup et al., 2021; Shi et al., 2011). Nevertheless, it is not known how biomarkers pass from central nervous system (CNS) to the saliva glands and finally into saliva. However, several theories have been proposed, including microfiltration, active or passive transport, or biomarkers being either expressed or produced by the saliva glands. It is also possible that biomarkers are excreted directly from degenerated axons or that the neurodegeneration that occurs in the nerve terminals alter the composition and production of saliva (Gleerup et al., 2019; Ashton et al., 2019). The aim of this study was to investigate saliva p-tau as a potential AD biomarker. Specifically, we examine the correlation of p-tau181 concentration in saliva with blood and CSF levels and evaluate its diagnostic performance across neurodegenerative dementias.

## 2 Methods

### 2.1 Ethics

This study was conducted between 20 March 2019 and 20 December 2019, at either the Copenhagen Memory Clinic, Copenhagen University Hospital, Rigshospitalet, or at the Regional Dementia Research Center, Zealand University Hospital, Roskilde. The project was approved by the Ethical Commitee of the Capital Region of Denmark (H-19000651) and the Danish Data Protection Agency (VD-2019-105), and all patients gave informed consent to participation.

### 2.2 Participants

A total of 125 participants with paired saliva (p-tau181), plasma (p-tau181) and CSF (Aβ42, p-tau181, t-tau) were included in this study. All patients underwent diagnostic evaluations, including standard blood tests, informant-based history, cognitive testing, lumbar puncture, structural scans [computed tomography (CT) or magnetic resonance (MRI)], and in most instances [^18^F]fluoro-deoxy-glucose-PET (FDG-PET) and neuropsychological testing.

Participants were distributed into three different groups: Cognitively unimpaired (CU) (*n* = 13), cognitively impaired with Aβ pathology (CI Aβ+) (*n* = 57) and cognitively impaired without Aβ pathology (CI Aβ–) (*n* = 55). Aβ positivity was locally established as CSF p-tau181/Aβ42 < 0.077. The CI participants consisted of AD dementia (*n* = 34), mild cognitive impairment (MCI, *n* = 36), alcohol-related dementia (ARD, *n* = 3), dementia with Lewy Bodies (DLB, *n* = 4), frontotemporal dementia (FTD, *n* = 7), progressive supranuclear palsy (PSP, *n* = 1), vascular dementia (VaD, *n* = 10), mixed dementia (*n* = 6), normal pressure hydrocephalus (NPH) (*n* = 6), other non-neurodegenerative disorders (*n* = 1), and dementias of unknown aetiology (*n* = 4).

Patients with AD fulfilled the NINCDS-ADRDA criteria for dementia due to AD, or MCI due to AD (McKhann et al., 1984). Patients with MCI due to other etiologies fulfilled the broad criteria of MCI suggested by the International Working Group in Mild Cognitive Impairment (Winblad et al., 2004). Patients with DLB fulfilled the fourth report of the DLB consortium criteria (McKeith et al., 2005), VaD fulfilled the Society for Vascular Behavior and Cognitive Disorders (VASCOG) (Sachdev et al., 2014), mixed dementia fulfilled both the NIA-AA and VASCOG criteria (McKhann et al., 1984; Sachdev et al., 2014). Patients with FTD fulfilled the criteria for the behavioral variant (Rascovsky et al., 2011), non-fluent aphasia or the semantic variant (Gorno-Tempini et al., 2011). Patients with NPH fulfilled the international guideline criteria for iNPH (Relkin et al., 2005), and patients with alcohol dementia fulfilled the International Statistical classification of Diseases and Related Health Problems 10th Revision (ICD-10) criteria (ICD-10 Version:2010, n.d.). The cognitively unimpaired (CU) did not fulfill any criteria for dementia or MCI.

### 2.3 Sample collection and measures of P-tau181

CSF, blood and saliva samples were collected from every patient approximately at the same time to avoid any diurnal variation. Participants were asked to desist from drinking, eating, or smoking for at least 30 min prior to saliva collection and to drink 100 mL of water just before the sampling. From 1 to 3 mL of unstimulated saliva for each patient were gathered in a 15 mL polypropylene tube. Plasma samples were collected in ethylenediaminetetraacetic (EDTA)-treated tubes. After the sampling, the tubes containing saliva, CSF and plasma were immediately placed on ice until they were centrifuged at 2000 g for 10 min at 4 °C and then stored at −80 °C until analysis.

Before testing, saliva samples were thawed on room temperature, vortexed at 2000 RPM for 30 s, centrifuged at 10,000 g for 10 min and diluted 20-fold with Tau2.0 buffer (Quanterix, Billerica, MA, United States). An ultrasensitive Single molecule array (Simoa) assay was used to measure saliva and plasma P-tau181 concentration. The scheme underlying the P-tau181 Simoa assay is based on the use of paramagnetic particles, called beads, that must be linked to specific target antibodies, in this case, mouse monoclonal antibodies specific to phosphorylated threonine 181 (AT270, Invitrogen). For the detection, a mouse monoclonal antibody, directed against the N-terminal region of tau (Tau12, Bio-Legend) and conjugated to biotin, has been used. A calibration curve has been created using a full-length recombinant tau1-441 phosphorylated *in vitro* by glycogen synthase kinase 3β (TO8-50FN; SignalChem, Vancouver, BC, Canada) and three quality control (QC) has been tested for checking the assay accuracy (Karikari et al., 2020). Total proteins in saliva have been quantified using the PierceTM BCA Protein Assay Kit (Thermo Fisher Scientific). The concentration of amyloid-42, T-tau and P-tau in CSF was defined using the INNOTEST enzyme-linked immunosorbent assays (Fujirebio, Ghent, Belgium).

### 2.4 Immunoprecipitation-mass spectrometry and immunohistochemistry

Please see Appendix 1 for the methods used in IP-MS analysis.

A set of de-identified leftover human salivary gland sections collected for diagnostic purposes at the Department of Pathology, Sahlgrenska University Hospital (approved by the regional ethics committee at the University of Gothenburg, #20140811) were treated with hematoxilin and eosin (H&E) stains. Another set of sections were stained with P-tau181 antibody. 5 μm-thick formalin-fixed paraffin embedded sections were sequentially treated with xylene twice for 3 min each, followed by xylene 1:1 with 95% ethanol for 3 min, 95% ethanol for 3 min, 70% ethanol for 3 min, and 50% ethanol for 3 min. Sections then underwent antigen retrieval in 10 mM sodium citrate buffer (pH 6) at 60 °C for 16–18 h. Sections were blocked with 3% H_2_O_2_ in 1X PBS for 10 min and non-specific binding was blocked for 90 min with 0.1% normal goat serum diluted in 1X PBS, followed by an overnight 4 °C incubation with the P-tau181 primary antibody (0.3 mg/mL; Invitrogen, ThermoFisher Scientific; Article#44-744). Sections were washed with 0.1% PBST and incubated with secondary antibody, (Alexa-flour647- Catalog# 1741783) for 60 min at room temperature. All the sections were then treated with autofluorescence quenching agent TrueBlack 1X for 30 s. Lastly, the sections were mounted with DAKO fluorescent mounting media and allowed to dry in the dark for 24 h for imaging.

### 2.5 Statistical analysis

Data were tested for normal distribution using the Shapiro–Wilk test. All saliva measures (total saliva protein, saliva P-tau181 and normalised saliva P-tau181) were not normally distributed (all, *p* < 0.001). This remained true when the data was log10-transformed, thus non-parametric tests were performed. Spearman’s rank correlation test was used to explore the association between saliva measures and the demographic information, and to evaluate also, the association with plasma and CSF measures. While the Mann–Whitney test was performed to evaluate significant differences between males and females, and between the amyloid positive group and amyloid negative group. Kruskal-Wallis test was used to investigate variations of the concentration of P-tau181 in saliva, plasma and CSF, in the different diagnostic groups. Statistical significance was established at *p* < 0.05. Statistical analyses were performed with IBM SPSS Statistics, version 27 (Armonk, NY, United States) and in GraphPad Prism (GraphPad Prism version 8.4.3 for Mac, GraphPad Software, San Diego, California, United States).

## 3 Results

### 3.1 Immunohistochemistry of salivary gland

**Figure 1:**
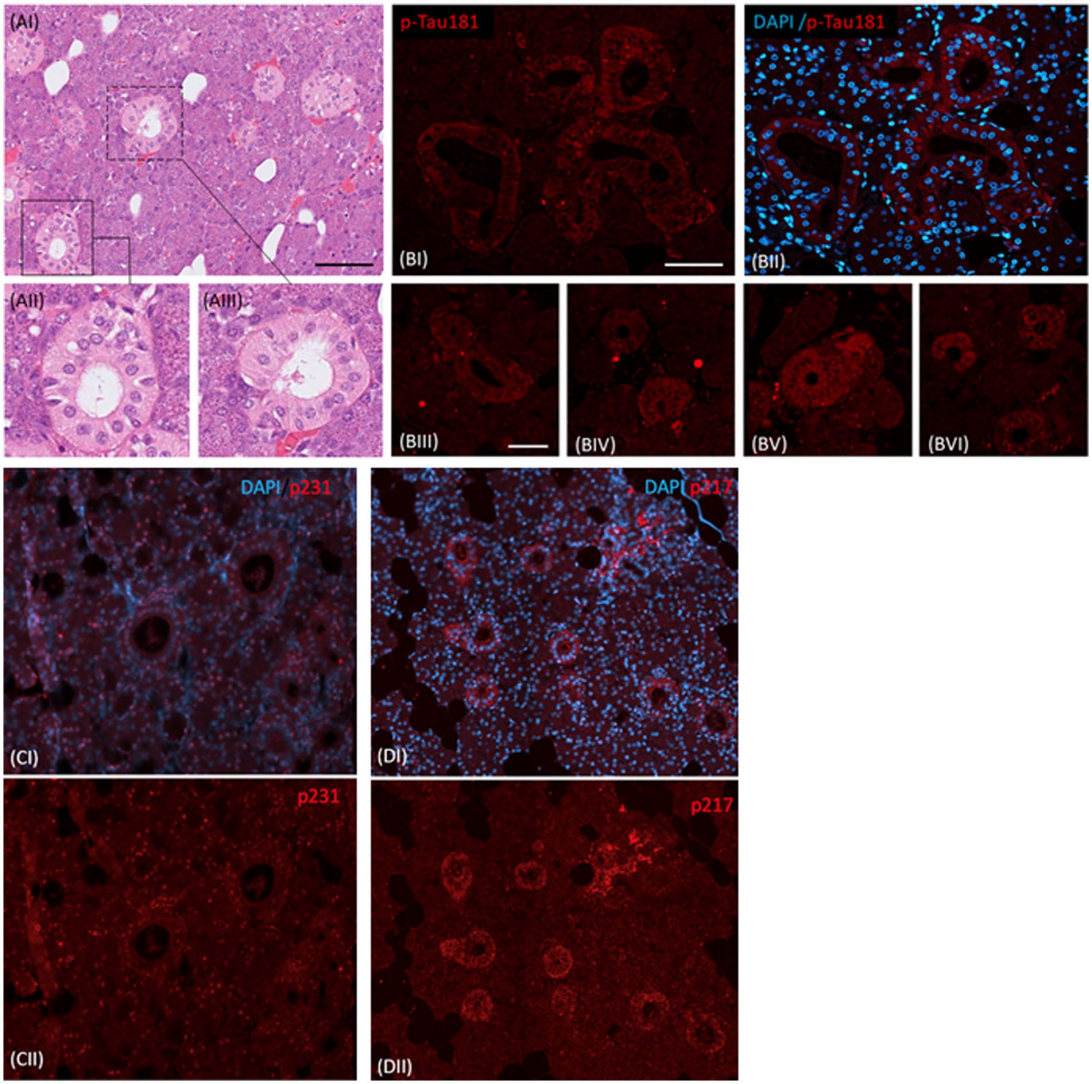
p-Tau181, p-Tau231, and p-Tau217 immunopositive striated ducts in saliva glands. **(AI)** Hematoxylin and eosin (H&E) stained region of saliva gland, with inserts **(AII-AIII)** depict striated ducts. **(BI)** p-Tau181 immunopositive striated ducts **(BII)** and a merge of p-Tau181 with nuclear stain (DAPI). **(BIII-VI)** depicts similar pictograms of the striated ducts detected across the region of saliva gland. Scalebar in (AI- 100μm; BI-50μm). **(CI)** merge of p-Tau231 straited ducts with nuclear stain (DAPI) and **(CII)** p-Tau231 immunopositive striated ducts. **(DI)** merge of p-Tau217 straited ducts with nuclear stain (DAPI) and **(DII)** p-Tau217 immunopositive striated ducts.

We first investigated the staining of saliva glands with antibodies targeting specific tau phosphorylations (p-Tau181, p-Tau231 and p-Tau217). In particular, p-tau181 indicated a subtle, yet distinct, granular accumulation of p-tau aggregates in the epithelial lining of the striated duct. Examination of staining patterns of the striated duct also indicate occasional dense p-tau181 positive granular accumulation. In addition, we confirmed staining patterns of both p-tau217 and p-tau231 (Figure 1).

### 3.2 Immunoprecipitation-mass spectrometry evidence for p-tau species in saliva

We then employed a recent multi-epitope IP-MS method tailored to blood (Montoliu-Gaya et al., 2023), in which we confirmed the presence of p-tau181, p-tau199, p-tau202, p-tau205, p-tau217 and p-tau231 in salivary fluid from pooled saliva (Supplementary Figure 1). We further investigated the specificity of the p-tau181 antibody used in the immunoassay analysis of clinical samples (see below). The detection of p-tau181 in saliva using this antibody was confirmed through untargeted MS, which identified the endogenous tau protein phosphorylated at Thr181 (Supplementary Figure 2).

### 3.3 Demographics of study participants

A total of 125 participants were included in the study (Table 1). The participants were classified as CU, CI Aβ + and CI Aβ- as described in the methods. Significant differences were found for age and sex between the groups. The total protein amount was measured in each saliva sample, which did not significantly change between groups (Table 1). A significant positive association between saliva total protein concentration and saliva p-tau181 was observed (*r* = 0.107, *p* = 0.014; Supplementary Figure 3) and therefore saliva p-tau181 values were also normalised by a ratio with saliva total protein concentration (saliva p-tau181^normalised^) in addition to the unadjusted saliva p-tau181 (saliva p-tau181^unadjusted^). Although age and sex differed significantly between group, there was no association between age or sex for saliva p-tau181^unadjusted^ or saliva p-tau181^normalised^.

**Table 1:** Characteristics of the study cohort.

	CU (*n* = 13)	CI Aβ+ (*n* = 57)	CI Aβ− (*n* = 55)	*p* value
Age, mean years (SD)	67.0 (8.3)	72.3 (7.8)	75.6 (7.0)	<0.001
Sex, F/M	3/10	34/23	17/38	<0.01
CSF Aβ42, pg./mL (SD)	1,087 (232)	697 (194)	978 (287)	<0.0001
CSF p-tau181, pg./mL (SD)	67.5 (15.8)	93.4 (44.6)	39.7 (14.9)	<0.0001
CSF t-tau, pg./mL (SD)	298 (157)	594 (263)	262 (112)	<0.0001
Plasma p-tau181, pg./mL (SD)	4.4 (2.0)	8.3 (5.5)	6.7 (4.1)	<0.01
Saliva p-tau181^unadjusted^_,_ pg./mL (SD)	238 (123)	362 (250)	357 (275)	0.41
Saliva p-tau181^normalised^ (SD)	0.47 (0.71)	0.51 (0.50)	0.51 (0.86)	0.45
Saliva total protein, μg/mL (SD)	885 (322)	915 (388)	938 (343)	0.86

Values are expressed as mean ± standard deviation (SD). All *p*-values were calculated by a one-way ANOVA or a Kruskal Wallis test, beside the *p*-value for sex, which was calculated by a Chi-squared test. *n*, number; M, male; F, female; CU, cognitively unimpaired; CI, cognitively impaired; CSF, cerebrospinal fluid; p-tau, phosphorylated tau; t-tau, total tau; Aβ, amyloid beta.

### 3.4 Plasma p-tau181, but not saliva p-tau181, is increased in cognitively impaired aβ + individuals

**Figure 2:**
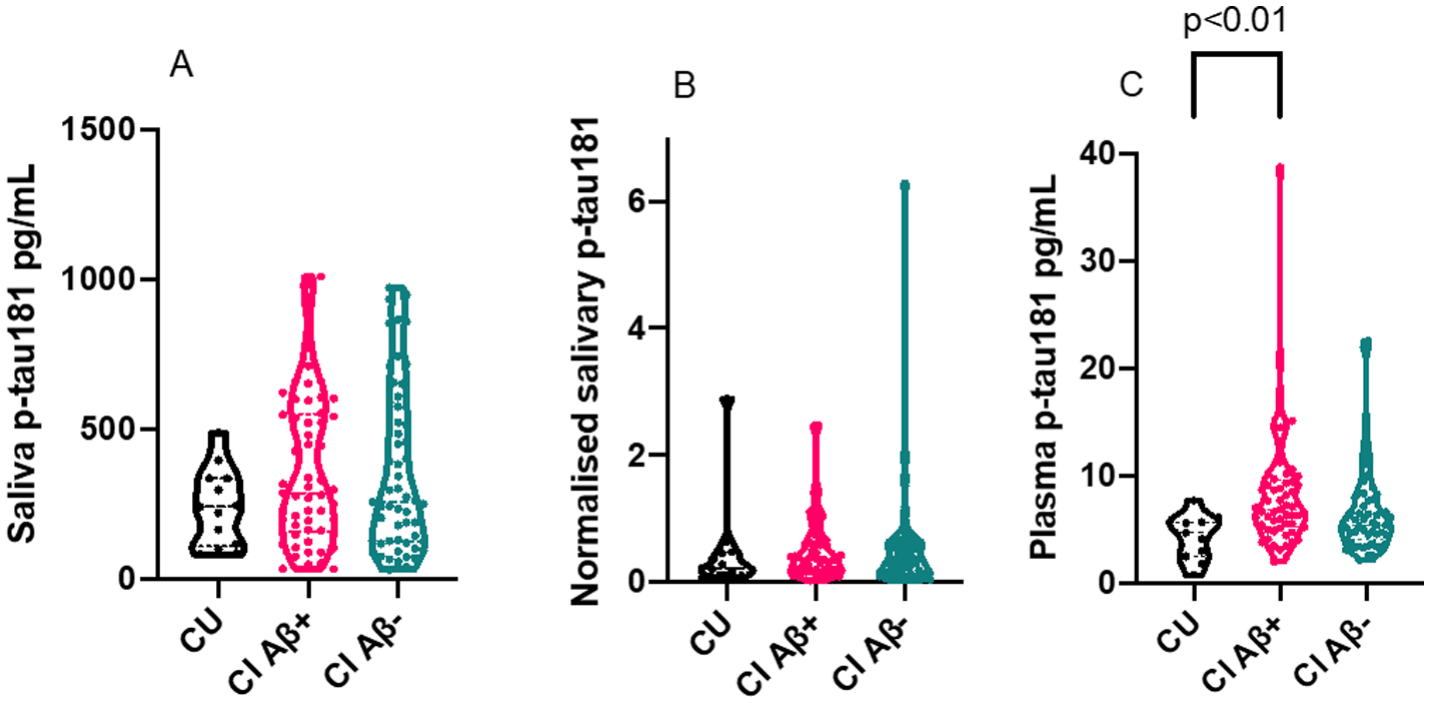
Violin plots of saliva p-tau181 and normalised saliva p-tau181. The figures show the biomarker results for CU (*n*=13), CI Aβ+ (*n*=57) and CI Aβ− (*n*=55). All *p*-values were calculated by a Kruskal Wallis test. **(A)** Truncated violin plot of saliva P-tau181 levels in CU, CI Ab+ and CI Ab−. The plot shows the median and interquartile range for each of the three groups. **(B)** Truncated violin plot of normalised saliva p-tau181 (saliva p-tau181/saliva total protein) levels in CU, CI Ab+ and CI Ab−. The plot shows the median and interquartile range for each of the three groups. **(C)** Truncated violin plot of plasma p-tau181 levels in CU, CI Ab+ and CI Ab−. The plot shows the median and interquartile range for each of the three groups. The plasma p-tau levels increased in CI Aβ+ individuals as compared to CU (*P* < 0.01). There was no significant change between CU and CI Aβ– (*p* = 0.2), and between CI Aβ+, and CI Aβ– (*p* = 0.06). CU, cognitively unimpaired; CI, cognitively impaired; p-tau, phosphorylated tau; Ab, amyloid beta.

Our results demonstrated that both saliva p-tau181^unadjusted^ and saliva p-tau181^normalised^ levels did not differ between CU, CI Aβ– and CI Aβ+ (Figures 2A,B). In contrast, and in the same participants, the plasma p-tau181 concentrations were significantly different between the groups (*p* < 0.01) (Figure 2C). As expected, the plasma p-tau levels increased in CI Aβ + individuals as compared CU (*p* < 0.01). There was no significant difference between CU and CI Aβ– (*p* = 0.2), or between CI Aβ+, and CI Aβ– (*p* = 0.06).

### 3.5 Saliva p-tau181 is not correlated to plasma or CSF biomarkers

In the whole cohort, there was no relationship between saliva p-tau181^normalised^ and plasma p-tau181 (*r* = 0.00078), CSF p-tau181 (*r* = 0.0036), CSF t-tau (*r* = 0.025) or CSF Aβ42 (*r* = 0.0071). These results did not change when examining the saliva p-tau181^unadjusted^ concentrations (Table 2). Interestingly, the relationship between saliva p-tau181 and CSF biomarkers improved when examining Aβ + patients in isolation. In this analysis the association between saliva p-tau181^normalised^ and CSF t-tau became statistically significant (saliva p-tau181^normalised^; *r* = 0.13, *p* < 0.01) (Table 2) (Supplementary Figure 4).

**Table 2:** The association between saliva p-tau181, plasma p-tau181 and CSF biomarkers.

	Whole cohort (*n* = 125)		Aβ + only (*n* = 57)	
	Saliva	Saliva	Saliva	Saliva
	p-tau181^unadjusted^	p-tau181^normalised^	p-tau181^unadjusted^	p-tau181^normalised^
Plasma p-tau181	*R*^2^ = 0.0082 (*p* = 0.32)	*R*^2^ = 0.00078 (*p* = 0.75)	*R*^2^ = 0.0023 (*p* = 0.72)	*R*^2^ = 0.0014 (*p* = 0.78)
CSF p-tau181	*R*^2^ = 0.0030 (*p* = 0.56)	*R*^2^ = 0.0036 (*p* = 0.53)	*R*^2^ = 0.00045 (*p* = 0.88)	*R*^2^ = 0.010 (*p* = 0.46)
CSF t-tau	*R*^2^ = 0.0043 (*p* = 0.49)	*R*^2^ = 0.025 (*p* = 0.093)	*R*^2^ = 0.0061 (*p* = 0.57)	*R*^2^ = 0.13 (*p* < 0.01)

Values are expressed as the coefficient of determination (R-squared) (*p*-value). *n*, number; CU, cognitively unimpaired; CI, cognitively impaired; CSF, cerebrospinal fluid; p-tau, phosphorylated tau; t-tau, total tau; Aβ, amyloid beta.

## 4 Discussion

The elevated level of p-tau in CSF, typically the phosphorylation on threonine 181 and more recently p-tau217 (Janelidze et al., 2020), is a validated diagnostic biomarker for AD. P-tau concentrations, in both plasma and CSF, increase only in the presence of cerebral Aβ burden (Kaeser et al., 2022). In this study, we confirmed the presence of p-tau epitopes in saliva and salivary glands by immunohistochemistry and IP-MS. We then measured the concentrations of p-tau181 in saliva and investigated their association with blood and CSF levels of p-tau181.

Our study found no significant difference of saliva p-tau181 concentration between the diagnostic groups, although a weak correlation between saliva p-tau181, normalised to the total saliva protein concentration, and CSF T-tau was found. Interestingly, this association was stronger and significant in the Aβ-positive patients. Another interesting aspect that emerged in our examinations, which confirms previous observations about saliva p-tau181, is the high saliva concentration relative to CSF and plasma values as seen in Table 1. This may point towards high molecular weight (HMW) tau, which includes the peripherally derived “big tau” being measured in saliva by the immunoassay employed in this study. A recent study demonstrated that low molecular weight (LMW) tau has low concentrations and greater specificity to AD (Janelidze & Ashton, in Press). Nevertheless, the presence of p-tau181, and other p-tau epitopes, in saliva was confirmed by the IP-MS.

In the past few years, saliva has been proposed to be a promising easily accessible matrix for biomarker quantification (Ng et al., 2024), and several studies show that many AD-related biomarkers [T-tau, P-tau (Shi et al., 2011), Aß peptides (Sabbagh et al., 2018), NfL (Gleerup et al., 2021), glial fibrillary acidic protein (GFAP) (Katsipis et al., 2021), acetylcholine (Ach) (Sayer et al., 2004), and lactoferrin (Gleerup et al., 2021; González-Sánchez et al., 2020; Ashton et al., 2021)] are quantifiable in saliva. One of the first studies that explored saliva t-tau with the Simoa technology revealed no differences between the diagnostic groups. Interestingly, t-tau was detectable in almost every participant with a concentration that was significantly higher than in plasma (Ashton et al., 2018). Meanwhile, the first results on saliva p-tau181 showed comparable levels to those observed in plasma (Marksteiner et al., 2022).

It is not yet clear how biomarkers pass from the central nervous system (CNS) to the saliva glands and finally into saliva, but some assumptions have been made. The main saliva glands, the submandibular, the sublingual and the parotid glands, have cholinergic innervation, specifically, from the glossopharyngeal and facial cranial nerve. Both of which are controlled by the autonomic nervous system (ANS). Since nerve terminals in the cholinergic system could be affected by degeneration in preclinical AD, this early deterioration could reveal why biomarkers are detected in saliva. It has been also proposed that biomarkers are excreted directly from the axons of the glossopharyngeal and facial cranial nerve, due to the position of their ganglions in the brainstem (Farah et al., 2018). Moreover, many blood-derived molecules, could simply move in saliva thanks to passive diffusion, active transport or microfiltration (Spielmann and Wong, 2011; Femminella et al., 2014). In addition, it cannot be ruled out that the expression and production of some AD biomarkers occur directly in the saliva glands which could express tau mRNA (Gleerup et al., 2019; Ashton et al., 2019; François et al., 2021). These results suggest that it is possible that there is a passage of brain p-tau to the saliva glands and then into saliva via the glossopharyngeal and facial cranial nerve and their ganglions in the brainstem. However, it is likely that the expression of tau mRNA directly in the saliva glands, misrepresents the concentration of the biomarker in saliva, rendering saliva p-tau181 not useful as an AD biomarker.

This study has some limitations. First, saliva biomarkers are still a new area of research, and therefore the optimal collection methods and preanalytical handling are not known (Ng et al., 2024). Second, a plethora of variables (oral hygiene, antidepressants, antipsychotics) might affect saliva production, flow rate and composition, and therefore future studies should take these factors into account (Einhorn et al., 2020). All included patients were recruited prior to diagnosis, and therefore they did not receive any antidementia medication, which could alter the saliva production (Miranda-Rius et al., 2015). Furthermore, the relatively small number of CU participants reduces the statistical power compared to the CI groups. However, this imbalance mirrors the real-world distribution of patients typically seen in memory clinics. Another limitation is the lack of CSF Aβ42 concentrations for some of the healthy controls that have been assumed to be amyloid negative and considered as such. In addition, saliva p-tau217 was not measured in the clinical samples despite the presence confirmed by IP-MS and IHC, which has been shown to outperform other p-tau biomarkers. Furthermore, this study might not be generalizable to other memory clinics since our memory clinic is also a tertiary referral centre with more complex cases. However, a strength of this study is also the external validity, and that the patients are a true representation of clinical cohort without selection bias. Furthermore, the validation of the biomarker findings using both immunohistochemistry and MS.

This study confirms the presence of phosphorylated tau epitopes in saliva and salivary glands using IP-MS and immunohistochemistry and demonstrates that p-tau181 can be quantified in saliva samples. However, saliva p-tau181 levels did not differ between diagnostic groups and showed only a weak correlation with CSF biomarkers, primarily in Aβ-positive individuals. The relatively high levels of salivary p-tau181 and the uncertain mechanisms behind its appearance in saliva suggest peripheral sources. Therefore, p-tau181 in saliva does not currently appear to be a reliable biomarker for AD. Nevertheless, the ability to detect AD-related biomarkers in saliva warrants further research into the origin, specificity, and technical handling of salivary proteins.

## Data Availability

The raw data supporting the conclusions of this article will be made available by the authors, without undue reservation.
