# Supplementary material for: Saliva phosphorylated tau concentration is not associated with Alzheimer’s disease, cerebrospinal fluid or blood biomarkers
Source: Front Neurosci. 2025 Dec 2;19:1718237. doi: 10.3389/fnins.2025.1718237 (PMC12705599; doi:10.3389/fnins.2025.1718237)
Supplement: Supplementary file 1 [file Data_Sheet_1.DOCX]

**Appendix 1**

**Targeted Immunoprecipitation-mass spectrometry for several tau peptides**

Saliva samples were analyzed with a previously described MS method [1,2]. Briefly, 1ml and 0.5ml saliva samples were vortexed at 2000 rpm for 30 seconds, and then centrifuged at 4,000 × g for 10 minutes. Tau protein was extracted through immunoprecipitation (IP) and perchloric acid precipitation, followed by solid phase desalting. Heavy-labeled AQUA peptide standards were spiked in during the sample preparation. The samples underwent digestion with trypsin for 18 h, after which they were lyophilized and stored at -80°C. Prior to LC-MS analysis, the samples were reconstituted in 50 µl of 0.05% trifluoroacetic acid and analyzed in single runs. The liquid chromatography and MS analysis settings matched those detailed in prior work [1,2].

******

**Figure 1: Levels of phosphorylated tau species p-tau181, p-tau217, p-tau231, p-tau199, p-tau202 and p-tau231 in saliva.**

The levels are depicted as the ratio of the endogenous tryptic peptide with spiked heavy labeled standards.

**Untargeted Immunoprecipitation-mass spectrometry to determine the specificity of the p-tau181 antibody in saliva**

Mass spectrometry preceded by immunoprecipitation (IP) was used to further explore the presence of p-tau181 in saliva. For the IP, tau181 antibody was added to magnetic Dynabeads M-280 and incubated for 2 hours at room temperature in a rolling mixer. The beads were washed three times with PBS, then the antibody was cross-linked with 20 mM dimethyl pimelimidate dihydrochloride (DMP) and 0.2 M triethanolamine (pH 8.2) and incubated for 30 minutes on the rolling mixer at room temperature. The reaction was stopped resuspending the pellet in 50 mM tris(hydroxymethyl)aminomethane (pH 7.5), followed by an incubation of 15 minutes. The cross-linked beads were washed twice in PBS and blocked with a solution of PBS and Roti Block. After 1 hour of incubation on a rocking platform at room temperature, the beads were resuspended in PBS and 8 mL of saliva were added. To obtain the binding of the antigen, an overnight incubation at 4 °C on a rolling mixer is needed.
The saliva sample was obtained by mixing 8 mL of unstimulated saliva from two healthy control subjects. The saliva sampling and processing followed the same protocol as the samples used for Simoa analysis, but no dilution was required in this case.
For the following steps, a magnetic particle processor (KingFisher, Thermo Fisher Scientific) was used to accomplish three wash steps of the solution. The first wash was performed with 1 mL of PBS 0.05% Triton-X 100, the second one with 1 mL of PBS and the third one, with 1 mL of 50 mM ammonium bicarbonate. As a last step in the magnetic particle processor, captured species were eluted in 100 µL 0.5% formic acid. The final solution was moved to a microcentrifuge tube and dried in a vacuum centrifuge. After this step, the sample was dissolved in 20 µL of 2.5 ng/µL trypsin (Trypsin Gold, Promega) in 50 mM ammonium bicarbonate incubated overnight at 37 °C. The following day, 2 µL of 10% formic acid in pure water was added to stop the reaction and thereafter the sample was dried in the vacuum centrifuge and stored at – 80 °C until analysis.

Nanoflow liquid chromatography (LC) coupled to electrospray ionization high resolution hybrid quadrupole–orbitrap tandem mass spectrometry (LC-MS/MS) was performed with a Dionex 3000 system and a Q Exactive (both Thermo Fisher Scientific, Inc.) in a similar way as previously published [3]. Briefly, the sample was redissolved in 7 µL 8% formic acid/8% acetonitrile in water. Using a sample loading buffer consisting of 0.05% trifluoroacetic acid/2% acetonitrile in water, 6 µL sample solution was loaded onto an Acclaim PepMap C18 trap column (length 20 mm, internal diameter 75 µm, particle size 3 µm, pore size 100 Å, Thermo Fisher Scientific, Inc.) for desalting and sample clean-up. Separation was performed by reversed-phase Acclaim PepMap C18 analytical columns (length 150 mm, internal diameter 75 µm, particle size 2 µm, pore size 100 Å, Thermo Fisher Scientific, Inc.). Separation was performed at a flow rate of 300 nL/min using a 50 min long linear gradient from 3% to 40% B. Buffer A was 0.1% formic acid in water and buffer B was 0.1% formic acid/84% acetonitrile in water. The mass spectrometer was operated in data dependent mode with higher-energy collision-induced dissociation (HCD) for ion fragmentation. Acquisition settings were: positive ion mode, resolution setting 70 000, 1 microscan, target values 10^6^, trap injection time 250 ms.

LC-MS/MS acquisitions were processed using Mascot Daemon v2.6/Mascot Distiller v2.6.3 (both Matrix Science) for charge and isotope deconvolution before submitting searches using Mascot search engine. The search was made against a custom-made tau-only database; for settings, see previous publications [3,4]


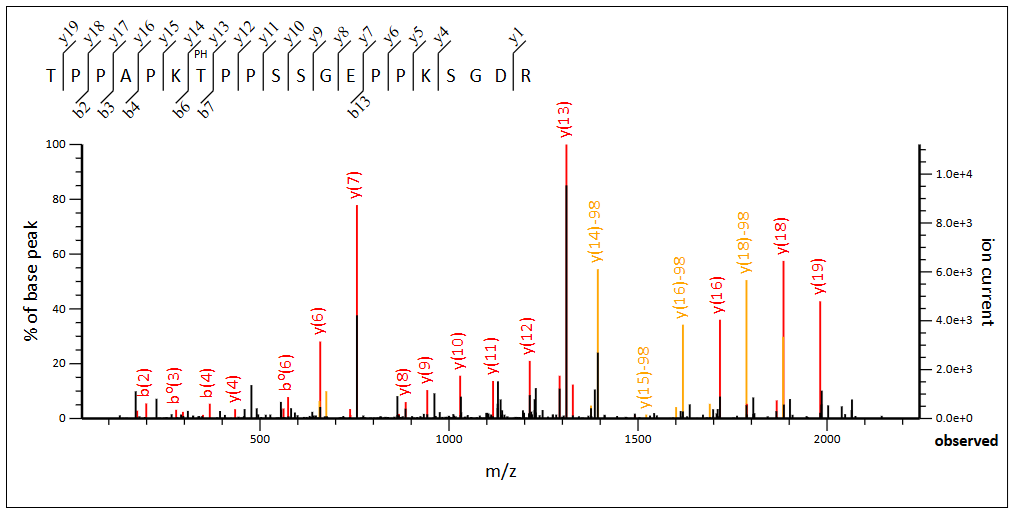


**Figure 2: Deconvoluted fragment ion spectrum of the tryptic tau peptide Tau175-194-p181, TPPAPK[pT]PPSSGEPPKSGDR, confirming the presence of p-tau181 in saliva.**

Precursor ion had an m/z of 695.00 and charge state 3+, and the obtained expect value from the search was 6.6 × 10^-12^. Matching peaks are in red and those representing neutral loss of H_3_PO_4_ (98 Da) in orange. Almost a full y-fragment ion series was obtained, unambiguously identifying the peptide and the phosphate group at Thr181.

**Figure 3: The association between saliva p-tau181 and saliva total protein**

The graph shows the relationship between p-tau181 and total protein in saliva for the whole cohort (r = 0.107, *P* = 0.014).

p-tau, phosphorylated tau

**Figure 4: The association between normalised saliva p-tau181 and CSF t-tau**

The graph shows the relationship between normalised saliva p-tau181 (saliva p-tau181/saliva total protein) and CSF t-tau for the whole cohort (r = 0.13, *P*<0.01).

t-tau, total tau

**References**

[1] Montoliu-Gaya L, Alosco ML, Yhang E, Tripodis Y, Sconzo D, Ally M, et al. Optimal blood tau species for the detection of Alzheimer’s disease neuropathology: an immunoprecipitation mass spectrometry and autopsy study. Acta Neuropathol 2024;147. https://doi.org/10.1007/s00401-023-02660-3.

[2] Montoliu-Gaya L, Benedet AL, Tissot C, Vrillon A, Ashton NJ, Brum WS, et al. Mass spectrometric simultaneous quantification of tau species in plasma shows differential associations with amyloid and tau pathologies. Nat Aging 2023;3:661–9. https://doi.org/10.1038/s43587-023-00405-1.

[3] Cicognola C, Brinkmalm G, Wahlgren J, Portelius E, Gobom J, Cullen NC, et al. Novel tau fragments in cerebrospinal fluid: relation to tangle pathology and cognitive decline in Alzheimer’s disease. Acta Neuropathol 2019;137:279–96. https://doi.org/10.1007/s00401-018-1948-2.

[4] Brinkmalm G, Portelius E, Öhrfelt A, Mattsson N, Persson R, Gustavsson MK, et al. An online nano-LC-ESI-FTICR-MS method for comprehensive characterization of endogenous fragments from amyloid β and amyloid precursor protein in human and cat cerebrospinal fluid. J Mass Spectrom 2012;47:591–603. https://doi.org/10.1002/jms.2987.
